# *MIDAS*: a methodological framework for high-speed high-energy diffraction microscopy data reduction. Part I: methodology

**DOI:** 10.1107/S2053273326004006

**Published:** 2026-05-28

**Authors:** Hemant Sharma, Jun-Sang Park, Peter Kenesei

**Affiliations:** a Advanced Photon Source Argonne National Laboratory 9700 S Cass Ave Lemont IL 60439 USA; Institute of Crystallography - CNR, Bari, Italy

**Keywords:** HEDM, high-energy diffraction microscopy, 3DXRD, 3D X-ray diffraction, *MIDAS* software, data reduction methodology, diffraction analysis, synchrotron radiation

## Abstract

The inversion of large-scale diffraction datasets from modern synchrotron sources presents a fundamental challenge in computational crystallography. This paper presents a unified algorithmic framework for the analysis of both near-field (morphological) and far-field (orientational and strain) high-energy diffraction microscopy (HEDM) data. We detail the mathematical formalisms and physical models that form the foundation of this methodology. Key aspects include a generalized model for detector distortion correction, robust algorithms for peak identification in noisy and overlapping patterns, a computationally efficient indexing formalism based on Friedel pair symmetry, and a decoupled iterative refinement scheme that exploits the differing sensitivities of position, orientation and lattice parameters to diffraction observables. We also describe the synergistic integration of near-field and far-field data streams, a critical feature of a truly comprehensive approach. The framework is validated in Part II of this series [Sharma *et al.* (2026). *Acta Cryst.* A**82**, 305–320] using both experimental Ti-7 Al datasets and synthetic reconstructions with known ground truth, achieving orientation accuracy of ∼0.05° and position accuracy of ∼10 µm on experimental data, and a 190× improvement in lattice parameter precision over conventional simultaneous parameter refinement on synthetic data. This integrated framework provides a powerful and extensible solution for turning raw diffraction images into actionable microstructural and micromechanical information.

## Abstract 2

This paper details the complete methodological framework implemented in the *MIDAS* software for processing high-energy diffraction microscopy (HEDM) data. We describe the specific algorithms, coordinate systems and physical models used for both far-field and near-field HEDM analysis. The framework is designed to handle the large and complex datasets from modern synchrotrons, enabling efficient and accurate extraction of microstructural information.

## 1. Introduction

The advent of high-brilliance synchrotron sources and fast area detectors has transformed diffraction science, but it has also created a fundamental challenge: the efficient and robust inversion of massive, complex, and often imperfect diffraction datasets into quantitative microstructural information. Solving this inversion problem requires a self-consistent and mathematically rigorous computational framework. This paper presents such a framework, designed to address this challenge in the context of high-energy diffraction microscopy (HEDM). HEDM is a term used broadly and often synonymously in the literature with 3D X-ray diffraction (3DXRD).

The HEDM technique has been applied to determine the grain-resolved 3D microstructure in a wide range of scientific problems, advancing the technique from a proof-of-concept stage (Poulsen, 2003 ▸; Poulsen, 2004 ▸; Suter *et al.*, 2006 ▸) to an established tool for materials science. This progress has been enabled by advances in both instrumentation (Park *et al.*, 2021 ▸; Shade *et al.*, 2015 ▸) and data reduction software.

Several grain-resolved diffraction-based microstructure imaging techniques have been developed over the years. These include diffraction contrast tomography (Ludwig *et al.*, 2009 ▸), point-focus HEDM (Bonnin *et al.*, 2014 ▸; Li *et al.*, 2023 ▸) and differential-aperture X-ray microscopy (Larson *et al.*, 2002 ▸) at the grain scale, and dark-field X-ray microscopy (Simons *et al.*, 2015 ▸) and Bragg coherent diffraction imaging (BCDI) (Maddali *et al.*, 2020 ▸) at the sub-grain scale. Among these techniques, HEDM offers a unique combination of fast data acquisition rates and the ability to investigate bulk-sized samples, often with higher levels of deformation. HEDM enables the simultaneous interrogation of large numbers of crystalline grains within a polycrystalline aggregate, with complete crystallographic orientations, 3D shapes and spatial locations deduced from comprehensive probing of each grain’s reciprocal space. HEDM is most often carried out in two modes: near-field (NF) HEDM and far-field (FF) HEDM. [High-resolution reciprocal-space mapping using a high-resolution detector placed far away from the sample is another modality of HEDM (Jakobsen *et al.*, 2006 ▸).] FF-HEDM often utilizes a slitted box beam and a medium-resolution detector placed at a large distance (∼1000 mm) from the sample, yielding grain-resolved crystallographic orientation, position and elastic strain tensor information in the illuminated volume of a polycrystalline material. In NF-HEDM, a 2D cross section of the sample is illuminated by a focused X-ray beam (Shastri *et al.*, 2007 ▸) and a high-resolution detector is placed at a small distance (∼10 mm) from the sample so that the crystallographic orientation variation within the grains and the grain shape information can be extracted from the diffraction patterns.

A number of data reduction approaches and software packages have been developed to obtain 3D maps from FF-HEDM and NF-HEDM datasets. These include *HEXRD* (Bernier *et al.*, 2011 ▸), *FABLE/ImageD11* suites (Wright, 2020 ▸), *IceNine/HEXOMPA* (Suter *et al.*, 2006 ▸), among others. This paper describes the data analysis framework implemented in the *Microstructure Identification using Diffraction Analysis Software* (*MIDAS*). The data analysis methodology can process FF-HEDM and NF-HEDM data individually or both datasets as an integrated package. For FF-HEDM, this framework builds on the analysis methodologies described previously (Sharma *et al.*, 2012*a* ▸; Sharma *et al.*, 2012*b* ▸) and incorporates several improvements to extend the range of materials that can be successfully studied using HEDM. For NF-HEDM, the framework utilizes FF-HEDM reconstruction results to speed up reconstruction times, similar to the approach presented by Nygren *et al.* (2020 ▸). This framework is unique in that it can leverage the results of either mode to speed up and improve the accuracy of the other. For example, while FF-HEDM indexing is routinely used to seed and accelerate NF reconstructions, the high-resolution spatial boundaries and intra-granular misorientation maps derived from NF-HEDM can conversely be fed back into FF-HEDM as highly accurate priors. This bidirectional feedback is particularly beneficial for resolving severely overlapping diffraction peaks in highly deformed materials or multiphase samples. Also, it has been developed to take advantage of large-scale supercomputers and graphics processing units (GPUs), thus enabling online analysis for real-time feedback during an experiment. A graphical user interface (GUI) for the framework was developed to allow easy determination of the instrument parameters necessary for HEDM data analysis. Once the instrument parameters are determined, the framework can be executed in single or batch analysis mode using configuration scripts with minimal GUI interaction to analyze large datasets automatically. Numerous scripts to post-process the results exist, serving as a bridge to software such as *DREAM3D* (Groeber & Jackson, 2014 ▸) and *Paraview* (Ahrens *et al.*, 2005 ▸) for microstructure and micromechanical state map visualization and information extraction. Analysis can be run on any system running LINUX, is open-source, and its code is publicly available for download at https://github.com/marinerhemant/MIDAS.

The framework also has a module for rapid µ-CT (micro-computed tomography) reconstruction using the GridRec algorithm (Marone & Stampanoni, 2012 ▸). These reconstructions can be used with FF-HEDM and NF-HEDM reconstructions to obtain a complete insight into the sample microstructure.

The paper is arranged as follows: Section 2 describes the geometry for HEDM experiments, Section 3 describes the methodology for FF-HEDM data reduction, Section 4 describes the methodology for NF-HEDM data reduction. Part II in this series (Sharma *et al.*, 2026 ▸) shows results obtained from the framework for both FF-HEDM and NF-HEDM techniques.

## 2. HEDM setup and geometry

### 2.1. HEDM setup overview

A typical HEDM experiment setup is an extension of the ‘rotating crystal method’ (Busing & Levy, 1967 ▸). A polycrystalline sample, mounted in a sample environment such as a load frame (Shade *et al.*, 2015 ▸) or a heat-treatment furnace (Sharma *et al.*, 2009 ▸; Pagan *et al.*, 2018 ▸), is rotated about an axis perpendicular to the incident monochromatic X-ray beam. (The rotation axis does not necessarily have to be perpendicular to the incident beam. However, ensuring that the rotation axis is perpendicular to the incident beam simplifies the mathematical description of the experiment. Practically, it also ensures that the same volume of material is illuminated by the incident beam during rotation and data acquisition.) The incident X-rays have calibrated wavelength based on X-ray absorption edges (Hubbell & Seltzer, 2024 ▸) and are highly monochromated (Shastri *et al.*, 2002 ▸; Shastri, 2004 ▸). As the sample is rotated, diffraction events from the crystallographic planes satisfying the Bragg diffraction condition are recorded on an area detector. Diffraction patterns are recorded at an angular interval (typically, the angular interval is 0.05–0.5°) chosen such that the spatial smearing of the diffraction spots on the detector during rotation is comparable with the spread of the peaks on the detector. For different HEDM modalities, detectors with various pixel resolutions are placed at different positions from the sample to acquire different features of the 3D microstructure.

FF-HEDM uses a medium-resolution detector (approximately hundreds of µm pixel pitch) placed hundreds to thousands of mm distance away from the sample. The center-of-mass (COM), average orientation and average complete elastic strain tensor of each grain in the illuminated volume are often the most common pieces of information that can be extracted from FF-HEDM. (Due to the large sample-to-detector distances, the diffraction signal is sensitive to the changes in crystallographic orientation and strain of a grain. The grain shape information cannot be resolved due to the detector position and resolution.) The relative grain size information is retained as the diffraction peak intensity. The incident beam shape that defines the field of view (FOV) ranges from a vertically focused (∼2 µm), horizontally slitted planar beam identical to the beam used for NF-HEDM to a vertically and horizontally slitted box beam. Typically, the horizontal dimension of the beam is set such that the sample volume of interest (VOI) is fully illuminated at all times during rotation. In cases where the VOI does not fit in one FOV horizontally (maximum is ∼2 mm), a data acquisition procedure using stitching can be used; a corresponding analysis workflow exists in the framework and the approach is described by Johnson *et al.* (2023 ▸).

NF-HEDM utilizes a high-resolution detector (∼1 µm pixel pitch) placed ∼10 mm away from the sample. A vertically focused, horizontally slitted planar beam is used for NF-HEDM to obtain a 2D map of the illuminated layer. Multiple sets of diffraction patterns are acquired at several sample-to-detector distances to utilize a triangulation-based reconstruction strategy. At each sample-to-detector distance, the sample is rotated 360° while acquiring diffraction patterns at the prescribed angular interval. Here, the diffraction signals from each grain are essentially a projection of the grain shape (due to the short sample-to-detector distances, the diffraction signal is sensitive to the grain shape and crystallographic orientation), but the signal is compressed in the vertical direction due to the small diffraction angles at high X-ray energies, leading to high uncertainty along the X-ray beam direction. Through sample rotation, multiple diffraction peaks (hence, multiple projections) from the same grain are acquired. These multiple sets of diffraction patterns are critical for improving the reconstruction accuracy. A space-filling orientation map of the illuminated volume is extracted from NF-HEDM. A 3D microstructure map is attained by translating the sample vertically and acquiring NF-HEDM data at the new layer. While the mathematical ray-tracing framework can technically support 3D box-beam illumination in the NF, restricting the incident illumination to a 2D planar section is typically required experimentally to achieve high-fidelity grain shape isolation. Because the diffraction signal on the high-resolution detector is essentially a parallel projection of the illuminated volume, utilizing a 3D box beam would result in the 2D projection of an entire 3D grain. This convolves the grain’s vertical (*z* axis) dimension with its in-plane morphology, creating significant ambiguity in resolving the true 3D shape boundaries. By focusing the beam into a 2D plane, the vertical position is strictly constrained by the beam thickness, ensuring that the detected diffraction spots are accurate planar cross sections of the grain. A highly accurate 3D morphology is then reconstructed by unambiguously stacking these isolated 2D layers.

Sub-grain techniques, including very far field HEDM (Jakobsen *et al.*, 2006 ▸), BCDI (Cherukara *et al.*, 2018 ▸) and DFXM (dark-field X-ray microscopy) (Simons *et al.*, 2015 ▸), use high-resolution detectors placed at several thousands of mm distance from the sample. These techniques can extract shape and intra-granular strain and misorientation fields of individual grains embedded in a bulk polycrystalline sample with tens of nm spatial resolution. The current version of the framework can provide critical information for the selection of interesting grains in a polycrystalline aggregate based on FF- or NF-HEDM and for the placement of high-resolution detectors (Maddali *et al.*, 2020 ▸) to realize seamless transition between grain-scale techniques (FF- and NF-HEDM) and these intra-granular scale techniques.

### 2.2. A generalized geometric formalism for HEDM

In the formulation used by this framework for a typical HEDM setup at the Advanced Photon Source (APS), the incident beam propagation direction and the rotation axis are critical in describing the setup. We denote the beam propagation direction as 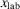. The rotation axis is established by the rotary stage in the HEDM setup, and it is denoted as 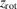; this is also denoted as ω rotation. The plane normal of the plane defined by 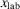 and 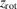 defines 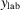. Finally, the cross-product between 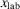 and 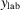 defines 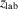. (In practical terms, the planar focused beam or the slit blades define the 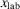
–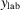 plane. The rotation axis is aligned to be perpendicular to the 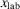
–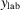 plane.) The intersection between 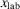, 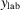 and 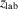 is denoted as 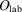. This formulation is consistent with and can be mapped to other common frameworks, such as that used in the *Fable* software suite (Oddershede *et al.*, 2012 ▸). The angle between 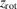 and 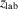 is denoted as the wedge or Ω. It is noteworthy that this framework accounts for a non-zero Ω, and it can analyze HEDM data acquired using such a setting (*e.g.* Park *et al.*, 2021 ▸). While an inclined axis geometry can be beneficial in certain cases, as shown by Kim *et al.* (2023 ▸), maintaining a perpendicular axis simplifies the experimental description and ensures a constant illuminated volume, which is advantageous for reconstruction. (The notation used in this article is summarized in Table 1 ▸ in Appendix *B*[App appb].)

**Figure 1:**
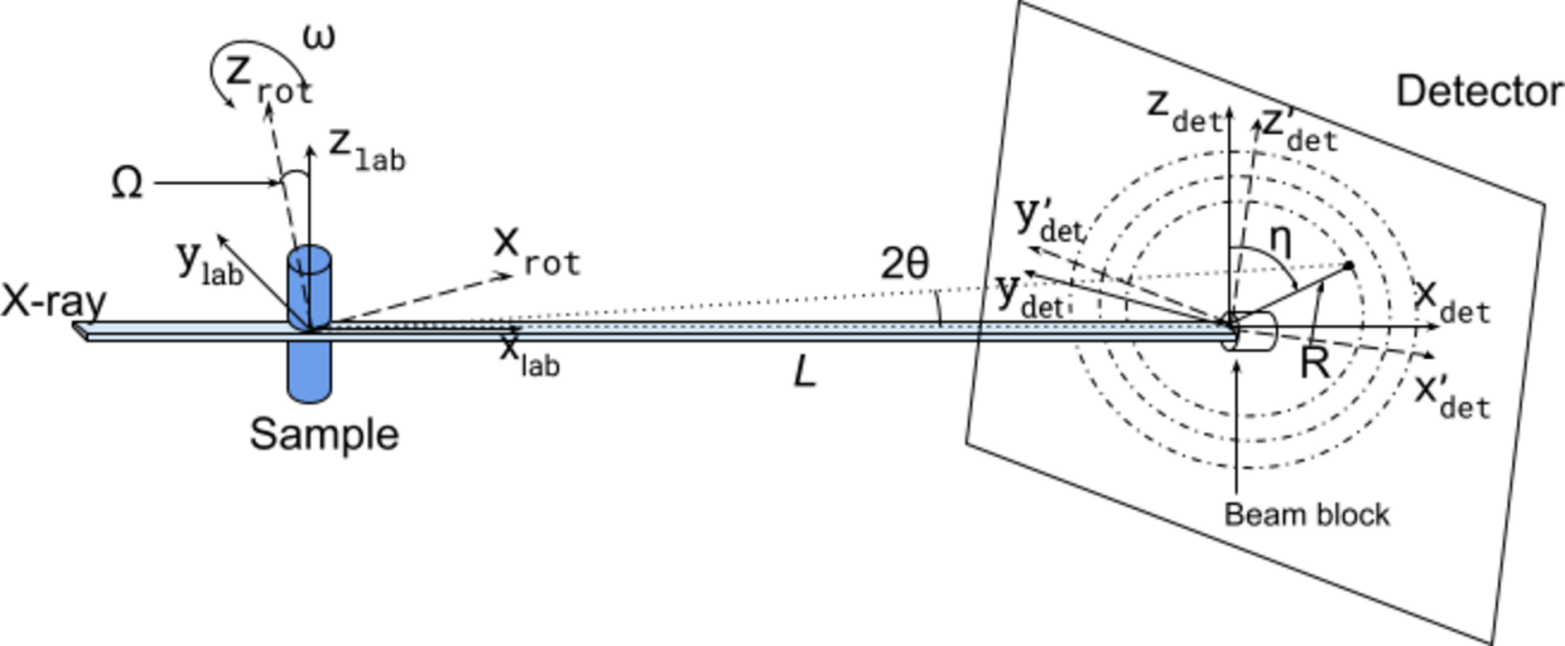
Experiment setup for FF-HEDM.

The diffraction events from crystallographic planes that satisfy the Bragg condition are recorded on an area detector as the sample is rotated about 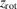. For FF-HEDM, the area detector and its pixel positions are described by three rotations 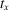, 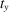 and 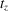, and three translations (these translations are often loosely referred to as the beam center and sample-to-detector distance) with respect to 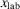, 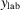, 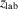 and 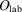, also shown in Fig. 1 ▸.

**Figure 2:**
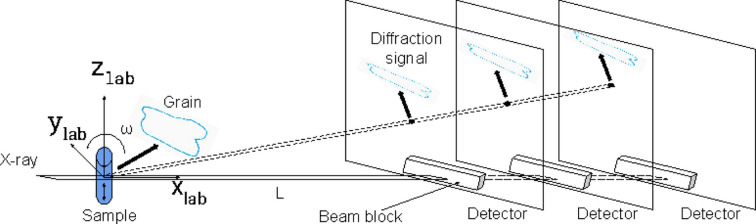
Experiment setup for NF-HEDM.

Diffraction data from two or more detector positions with the same sample position and rotations are acquired in NF-HEDM, shown in Fig. 2 ▸. This allows for ray-tracing for the same diffraction peak to obtain high-resolution microstructure information. The area detector is typically translated along *x* with minor adjustments in the *y* and *z* directions. The translations result in a set of beam-center and sample-to-detector distances for each detector position. The three rotations 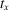, 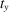 and 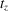 are assumed to be the same for all detector positions.

It is important to note here that the *sample* coordinate system is not intrinsic to the measurement: the experimenter needs to track the alignment of the *sample* with respect to the *laboratory* coordinate system and properly translate/rotate/align the two coordinate systems to perform meaningful material science experiments.

## 3. FF-HEDM data reduction procedure

This section describes the typical procedure used to reduce a FF-HEDM dataset.

### 3.1. A mathematical model for experimental geometry and detector distortions

Experimental setup parameters are determined using stress-free reference materials such as CeO_2_ or LaB_6_ powder and single-crystal ruby (Wong-Ng *et al.*, 2001 ▸) whose lattice parameters are well known. Here, we provide a short summary of this step. Readers are referred to the works of Sharma *et al.* (2012*a* ▸), Borbely *et al.* (2014 ▸) for details.

Using a set of powder reference diffraction patterns acquired at ω angles separated by 180°, rotation-axis-to-detector distance (*L*) and the projection of 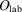 to the detector (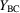, 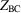), tilts of the detector (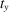, 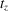) and spatial distortion field parameters of the area detector are determined. The tilt of the detector about the X-ray beam (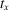) and wedge angle cannot be determined by the powder reference patterns.

The initial guesses for *L*, 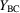, 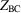, 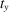, 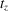 and spatial distortion field parameters are obtained using a GUI component of the *MIDAS* software package (MIDAS-FF-GUI) and matching the simulated diffraction patterns to the measured counterpart. A least-square fit to minimize the difference between the expected radius (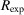) and the observed radius (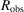) along the azimuth (η) is then used to determine the experiment parameters. The expected radius 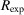 is calculated as
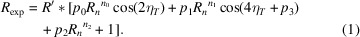
This is an extension of spatial distortion for the GE-41RT detector described by Lee *et al.* (2008 ▸) and has been shown to work for generic area detectors. Here, 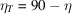 and 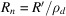. 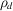 is the maximum radius over which the distortion function is valid, 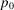, 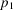, 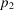, 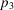, 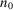, 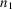 and 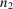 define the spatial distortion and are determined by fitting diffraction data from the reference. Typical values for the exponents, 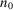, 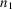 and 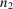 are 2, 4 and 2, respectively. 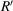 is the tilt-corrected 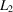 norm of the diffraction peak from 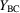, 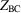.

Equation (1) serves as a parametric model for the detector’s non-linear spatial distortion, which arises from imperfections in the hardware, such as fiber-optic taper alignment or sensor-to-sensor mounting variances. The coefficients (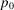, 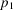, 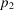) act as scaling factors for the radial and azimuthal components of the distortion field, while the exponents (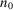, 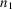, 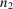) account for the non-linear coupling between the detector plane geometry and the incoming diffraction signal. By mapping the observed peak positions (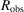) to their expected positions (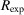) across multiple reference powder diffraction rings, we solve for these coefficients via least-squares minimization. This effectively ‘unwarps’ the detector image, ensuring that the coordinate system accurately reflects the physical reciprocal space.

For multi-module detectors such as the DECTRIS Pilatus, where inter-module gaps and minor misalignments are present, the framework incorporates a specific calibration routine. This routine uses data from multiple detector translations to precisely map the position of each module, similar to the technique described by Wright *et al.* (2022 ▸). This allows for the reconstruction of a seamless diffraction pattern prior to peak searching, correcting for module displacements that are typically on the sub-pixel level.

A set of FF-HEDM data from a single-crystal reference determines 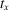 and Ω. Here, the parameters determined using the powder reference patterns are used as the initial guess and optimized with 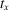 and Ω. In case a single-crystal reference is used, all the parameters of the setup (including 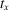 and wedge) are optimized together with the crystal parameters (lattice parameter, crystallographic orientation and COM position) using a full FF-HEDM dataset to characterize the experimental geometry fully. 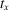 and wedge can only be determined using single/polycrystal diffraction using characteristic diffraction peaks from a user-selected grain.

### 3.2. Peak search

FF-HEDM conventionally relies on the COM position of the diffraction peaks to extract the COM, orientation and strain of the grains. To determine the COM of the diffraction peaks, a 2D peak shape optimization algorithm is used, which can fit multiple overlapping peaks simultaneously. Readers are referred to Sharma *et al.* (2012*a* ▸) for details; a brief overview is provided here.

For deformed materials with large intra-grain strain/mosaicity, diffraction peaks are smeared in 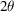, η and ω directions. The framework treats the diffraction images in parallel to reduce computation complexity, first computing the COM of each diffraction peak in 2D (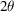 and η). The diffraction peaks stretch along the azimuthal direction for large deformations or significant overlaps. Calculating a COM for such deformed peaks in detector coordinates (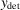, 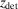) would not yield accurate results because of the significant curvature. Thus, the pixel positions in a diffraction image are first transformed from 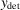, 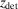 to diffraction and azimuthal angles (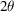, η). A threshold filter is applied to remove background intensity after subtracting electronic noise (dark-field signal) from this polar image. Alternatively, the user can choose which diffraction planes to include in the analysis and variable background for each plane. If the user selects to process certain diffraction planes on the detector images, a small area of interest is constructed by excluding all the pixels outside a small ring centered around each selected Debye–Scherrer ring on the detector. A connected component labeling step identifies regions with positive signals. Afterwards, a watershed algorithm searches for a guess of the number of overlapping diffraction peaks in each connected region. This step also yields a guess for the COM position of each diffraction peak and the pixels belonging to it. An optimization algorithm then optimizes the COM of the peaks identified in the previous step by fitting the peak shapes to a shape function (2D pseudo-Voigt is commonly used). An intensity-weighted COM is calculated for peaks present in multiple exposures during rotation. In the end, diffraction peaks are characterized by total integrated intensity, *I*, COM, and corresponding widths along 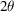, η and ω directions. Depending on the computation resources available, each region detected during connected component labeling on each diffraction image can be processed in parallel.

Recently, the framework has been extended to use a machine learning based neural network, *BraggNN*, to determine the characteristics of diffraction peaks significantly faster than peak shape optimization. *BraggNN* has been demonstrated to result in grain characteristics up to 15% more accurate than the peak shape optimization algorithm (Liu *et al.*, 2022 ▸).

### 3.3. Transform

The diffraction plane normal, or g-vector 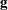, specified by Miller indices (*hkl*), is defined as the vector normal to the diffracting crystallographic planes (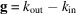). In our laboratory coordinate system, the components of a measured 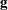 are determined from the corrected diffraction angles (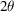, η) and the sample rotation angle (ω). Diffraction peaks that span multiple consecutive ω frames (*i.e.* are present in more than one detector image due to the finite angular width of the diffraction event) are merged to generate 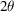, η and ω centroid positions for each peak. After correcting for detector tilts and distortion, the centroid position of each diffraction peak is computed in the laboratory coordinate system (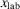, 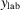, 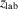) and a corresponding ω. In the case of small exposure times and detectors with serialized read-outs, effective exposure of individual pixels falls to different points of time, hence different pixels of the detector are associated with different time windows and consequently corresponding ω centroid position (Lee *et al.*, 2008 ▸). This, combined with continuous acquisition typically used during an HEDM experiment, requires correction for the ω position for each diffraction peak depending on its original location on the detector with respect to the hardware-based read-out sequence. The GE-41RT detector used at beamline 1-ID of the APS has a read-out starting from the center of the detector and going towards the vertical edges of the detector (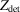). The ω correction is carried out as follows: 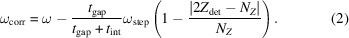
Here, 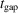 is the detector read-out time, 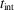 is the integration time, 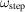 is the ω interval for each diffraction image, 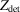 is the vertical position of the diffraction signal, 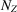 is the number of vertical pixels on the detector. This correction is negligible in cases where 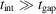. Because the detector read-out is serialized in the vertical direction while it is working in parallel in the horizontal direction (assuming the typical orientation of the detector) for the top and bottom half of the panel independently, different pixels are read at slightly different times relative to the sample’s angular rotation. The parameter 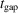 represents the fixed read-out time for a half-frame, and 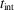 is the actual integration time (while the read-out is suspended) for each frame. During continuous rotation at a constant rate 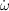, a diffraction signal arriving at a vertical pixel position 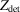 is recorded at a specific moment in time within the integration window. Equation (2) corrects the resulting azimuthal ‘smearing’ by shifting the recorded rotation angle ω based on the time elapsed from the start of the detector frame read-out to the arrival of the signal at 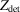, effectively referencing all signals to the center of their effective exposure intervals.

By calculating the powder diffraction intensity (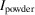), each diffraction peak is assigned an equivalent grain volume as follows (Offerman & Sharma, 2010 ▸): 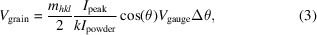
where 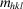 is the multiplicity of the *hkl* plane, θ is the Bragg diffraction angle, 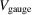 is the illuminated volume defined by the intersection of the X-ray beam and the sample, *k* is the number of diffraction images in which the diffraction peak is present, 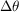 is the rotation angle swept by the diffraction peak, described as (Sharma *et al.*, 2012*b* ▸): 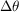 = 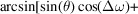

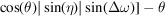
. This 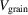 is used later during indexing (Section 3.4) to filter out mismatching peaks (peaks belonging to the same grain must have similar 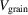) and during grain determination (Section 3.6). In case the X-ray beam is focused in the vertical direction, equation (3) is modified as
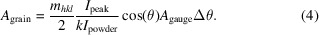
Grain radius from 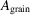 is calculated assuming circular discs or from 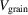 assuming spherical grain shape.

Diffraction peaks, now characterized by 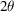, η and ω, are used to compute 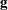 assuming the diffracting grain is located at 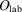. These 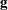
’s are provisional and are updated during the indexing and refinement stages once the grain’s true position is computed.

The *SGInfo* (Grosse-Kunstleve, 2023 ▸) package is used to compute valid *hkl* planes for the crystal structure under investigation. Diffraction rings are defined as the group of *hkl* planes sharing the same 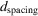. [Note here that (potentially) different *hkl*’s with the same 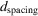 are considered the same diffraction ring.] Diffraction peaks are assigned to individual diffraction rings according to the 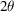 value of the peak (it must be within 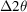 from the ideal 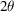). In cases where the difference in ideal 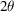 of multiple rings is smaller than 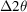, the same diffraction peak is assigned to multiple rings. (The framework is very robust in filtering out these misassigned peaks. This is demonstrated in Part II of this series.) The framework allows the user to select which diffraction rings and *hkl* planes to use during the analysis.

### 3.4. An indexing formalism based on Friedel pair constraints

Indexing is the process of assigning diffraction peaks to individual grains. Indexing in the framework is divided into the following steps: identifying candidate orientations with a high likelihood of being present in the sample, forward simulation of diffraction from the candidate orientation, and matching with observed data.

We start with the problem of identifying the candidate orientations. As described earlier (Sharma *et al.*, 2012*b* ▸; Bernier *et al.*, 2011 ▸), using diffraction data to reduce the orientation search space is computationally advantageous. For example, the position of a diffraction peak in 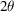, η and ω can be used to determine the normal to the originating *hkl* plane (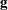). 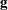 is defined as the vector normal to the diffracting crystallographic planes, with a magnitude of 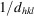. The only unknown factor in determining the full crystallographic orientation of the grain is the in-plane rotation of the (*hkl*) plane. The framework uses this information by selecting a subset of diffraction peaks, typically peaks on one of the diffraction rings, then carrying out the full forward simulation with different rotations of the *hkl* plane and choosing the orientation with the highest match with observed data. This way, in case multiple starting diffraction peaks are selected from each grain, each grain is independently determined multiple times. This is a figure of merit (called *MinNrSols*). (The framework does not filter diffraction peaks as they are assigned during indexing: the same diffraction peak can be assigned to multiple grains. This has been proven advantageous in cases where diffraction peaks from multiple grains overlap.) *MinNrSols* is a user-defined integer threshold. It represents the minimum number of independent solutions, each derived from a different starting diffraction peak, that must agree on a grain’s orientation and position within a given tolerance before it is accepted as a valid candidate. This provides a powerful filter against false positives that can arise from chance matches in dense patterns.

**Figure 3:**
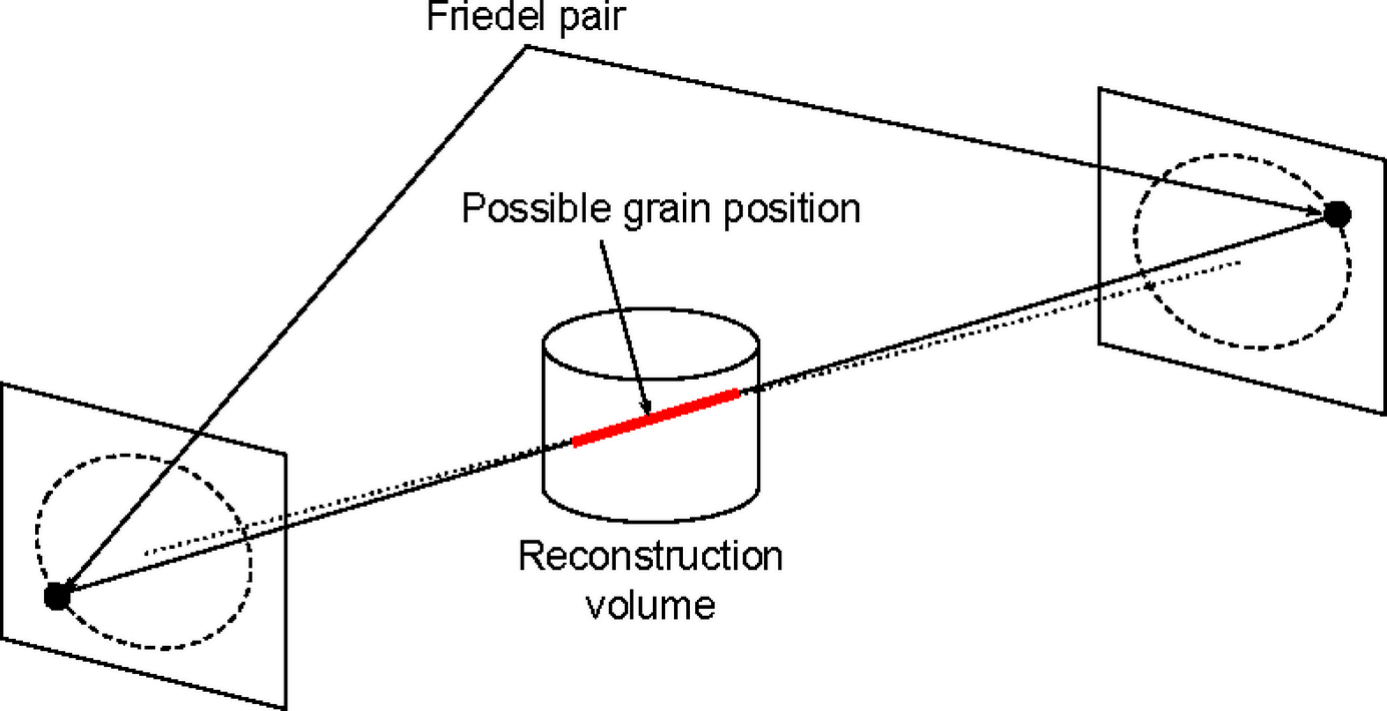
Schematic showing Friedel pairs. The sample is considered stationary while the whole experimental setup is rotated by 180°.

One issue with the approach described above is that in case the diffracting grain is not present at 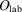 or 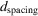 of the *hkl* plane is different from the theoretical value due to crystallographic strain, diffraction peaks are not present at the ideal position on the detector. This leads to an error in the diffraction plane’s calculated direction, leading to many false matches when combined with a high density of diffraction peaks on the diffraction rings (Sharma *et al.*, 2012*b* ▸). One way to solve this is to try forward simulation with multiple positions of the grain in the reconstruction space (imaginary sample) and multiple 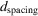 values for the starting *hkl* plane. However, this can quickly become a very compute-intensive problem. Here, the framework uses experimental information by forming Friedel pairs (Moscicki *et al.*, 2009 ▸) (same *hkl* plane, but diffraction in the reverse direction or from the other side of the plane)^1^ to significantly speed up the computation. In case two diffraction peaks form a Friedel pair, the intersection of a virtual line between the two and the reconstruction space fixes the position in the sample where the diffracting grain can be present and 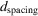 of the *hkl* plane corresponds to the Friedel pair. Utilizing this information for each possible Friedel pair, the framework reduces the grain position search space in the sample to a line. This is shown schematically in Fig. 3 ▸.

Once a candidate orientation is selected, forward simulation involves calculating the expected position of the diffraction signal for each user-selected *hkl* plane, described by Sharma *et al.* (2012*b* ▸). To speed up the comparison with observed data, the framework pre-computes sparse-matrix look-up tables (LUTs) for each diffraction ring and these are stored and accessed as shared-memory arrays across all CPU cores on a computation node. These LUTs map the observed 3D position of diffraction peaks to a regularized 3D sparse matrix, which reduces the computation requirement for searching through observed data by multiple orders of magnitude. Only diffraction peaks with similar originating grain sizes can be matched,^2^ which is a user-selectable tolerance parameter. Due to the finite sampling of the continuous position, orientation and strain parameter spaces, tolerances are used when comparing simulation with experiment data to account for experimental errors. The combination of grain position, crystallographic orientation and lattice parameter yielding the highest fraction of match with observed diffraction peaks (*Confidence*) and lowest difference in observed and simulated position of diffraction peaks (using angles between 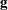
’s) is selected as the best candidate grain for the next step. A key figure of merit for a candidate grain, *Confidence*, is defined as the number of experimentally observed peaks matched to that candidate to the total number of peaks theoretically expected for that grain’s orientation within the measured experimental range. 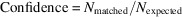.

In practice, the Friedel-pair-constrained candidate generation reduces the effective orientation/position search space by a factor of 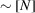 compared with a brute-force grid search over the full sample volume, enabling indexing of 200+ grain aggregates in under 10 s on modern CPU hardware.

### 3.5. Refinement

To address the high-dimensional and tightly coupled nature of the grain parameter refinement problem, we introduce a decoupled iterative optimization scheme. This strategy sequentially optimizes subsets of parameters based on their differing sensitivities in reciprocal and rotational space, leading to more stable and rapid convergence.

The position (3 values), crystallographic orientation (3 values) and lattice parameters (6 values) of each candidate grain are refined in parallel using the indexed values as an initial guess. The placement of the detector far away from the sample has an undesirable consequence: the sensitivity of diffraction peak position to changes in grain position, crystallographic orientation and lattice parameter varies widely, and consequently a generalized optimization using a single function is error-prone. An innovative iterative optimization routine is used to refine the grain’s position, orientation and lattice parameters, taking advantage of the different sensitivities of the diffraction peak positions (on the detector and in ω) to these parameters:

(i) Position of the grain does not affect the ω position of diffraction peaks.

(ii) Lattice parameter has a smaller effect on the ω position than the detector position of diffraction peaks.

(iii) Crystallographic orientation significantly affects the ω position and the detector position of diffraction peaks.

**Figure 4:**
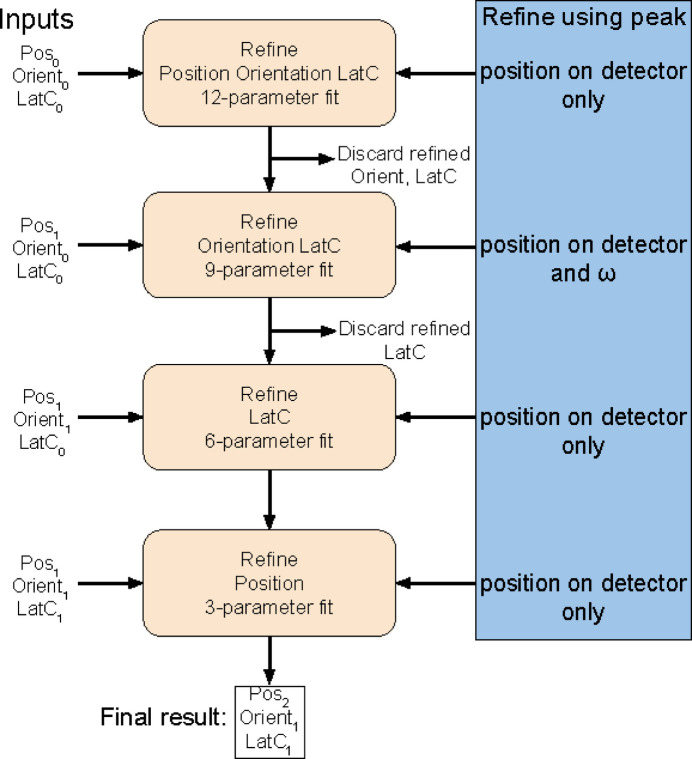
Workflow describing the optimization routine to compute the best center-of-mass position (Pos), crystallographic orientation (Orient) and lattice parameter (LatC) for each grain in *MIDAS*. At each step, the characteristics of diffraction peaks used for optimization are shown in the blue box.

The optimization routine used in the framework is shown schematically in Fig. 4 ▸. The routine starts with a 12-parameter fit (step 1, the first orange refinement box in the figure) using the 2D position of diffraction peaks on the detector (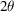 and η). The optimized orientation and lattice parameter from this step are discarded. Using the newly obtained position, a 9-parameter fit (step 2) optimizing the orientation and lattice parameter is carried out using the 3D position of diffraction peaks (minimizing the angle between the expected and observed diffracting plane normals). The optimized lattice parameter from this step is discarded. In the next step, using the optimized position from the first step and the optimized orientation from the second step, a 6-parameter fit (step 3) is carried out to optimize the lattice parameter using the 2D position of diffraction peaks on the detector (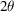 and η). Using optimized position, orientation and lattice parameter from the three fitting steps, the position is optimized again using a 3-parameter fit (step 4) by minimizing the difference in expected and observed 2D position of diffraction peaks (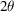 and η). Final grain properties are position, orientation and lattice parameter obtained from steps 4, 2 and 3, respectively. At each step, the corrections for detector tilts, wedge and spatial distortion of the detector are recomputed and applied to the observed diffraction peaks according to the then-determined grain position. It should be noted that the corrections for detector tilts, wedge and spatial distortion are global apparatus descriptors. Consequently, these parameters are not optimized for each grain independently; rather, they are refined simultaneously against all diffraction peaks from a global set of user-selected reference grains distributed throughout the sample volume. This ensures the geometric model remains self-consistent across the entire experimental setup.

Optimization in the framework is carried out using the *NLopt* library (Johnson, 2023 ▸). The quantitative benefits of this decoupled iterative scheme are demonstrated in Part II of this series (Section 2.3.3), where we show using synthetic ground-truth data that it achieves a factor of 190× improvement in lattice parameter precision compared with simultaneous optimization of all 12 parameters, while also being computationally faster.

### 3.6. Grain determination

The procedure described in *Indexing* (Section 3.4) and *Optimization* (Section 3.5) ensures that multiple independent solutions to each grain are determined. The next step filters outliers and false matches. Using a criterion determining the minimum number of independent solutions to a grain (*MinNrSols*),^3^ the framework will determine the successfully identified grains. There are multiple criteria to determine whether two solutions belong to the same grain: crystallographic misorientation between the solutions must be lower than 0.01°, the position of the solutions must be within 15 µm and at least 90% of diffraction peaks assigned to each solution must be shared. At this stage, the framework will further filter out any mis-indexed peaks and run optimization described in Section 3.5 to arrive at the final descriptors for each grain. The size of each grain is calculated as the mean of sizes from all the indexed diffraction peaks.

In the special case of twin orientations, some diffraction peaks of the twin grains overlap, and the user can decide to use one of two paths:

(i) Choose to analyze as is, assigning the overlapping diffraction peaks to all the twin grains. Here again, there are two choices: selecting a tight tolerance for diffraction peak matching based on the originating grain size will filter out all the overlapping peaks during indexing, and selecting a larger tolerance will include the overlapping peaks but will affect the grain size calculation after indexing.

(ii) Merge the twin grains: sometimes it might be interesting to merge the twin grains (*e.g*. when calculating parent grain size). In this case, the framework can use the twin orientation relationship (user selectable)^4^ to merge all the grains and diffraction peaks which share the twin orientation relationship. The framework calculates the overlapping and non-overlapping peaks and will only qualify twins if the grain size calculated from overlapping peaks is within 5 µm of the sum of grain sizes calculated from non-overlapping peaks.

For materials like Cu and Ni that form large twin-related domains (TRDs) with 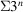 relationships, the framework can handle multiple generations (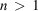) either by sequential matching or by expanding the user-defined twin orientation relationships.

### 3.7. A framework for crystallographic determination

The framework provides two distinct methods for calculating the crystallographic strain for each grain. The choice of method depends on the scientific goal.

*Lattice parameter refinement*. This method maps the optimized lattice parameters (*a*, *b*, *c*, α, β, γ), representing the unit-cell edge lengths and inter-axial angles, to the ideal, unstrained lattice parameter (Sharma *et al.*, 2012*b* ▸). This method is generally recommended for determining the average strain state. The strain tensor in the laboratory coordinate system is calculated as
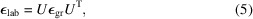
where *U* is the orientation matrix defining the grain-to-laboratory coordinate system rotation. 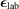 is the strain in the laboratory coordinate system. If the sample is not aligned with the laboratory coordinate system at 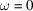, the orientation from the framework (in the laboratory coordinate system) must be rotated to the sample coordinate system by updating *U* accordingly. 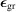 is calculated using the optimized lattice parameters (*a*, *b*, *c*, α, β, γ) as 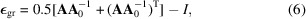
where *I* is the identity matrix, 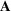 is defined as
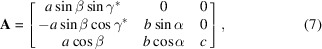

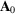
 is equation (7) for undeformed lattice parameters and 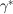 is γ in reciprocal space. Matrix **A** defined in equation (7) facilitates the mapping between the physical unit-cell parameters (*a*, *b*, *c*, α, β, γ) and an orthonormal basis. The transformation accounts for the tilt and obliquity of the unit-cell edges, effectively ‘orthogonalizing’ the crystallographic space so that it can be directly related to the laboratory Cartesian coordinate system. This is a necessary mathematical prerequisite for calculating the strain tensor in the laboratory frame using the identity matrix *I* as a reference for the undeformed state. The reciprocal-lattice parameters (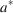, 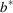, 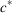, 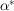, 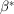, 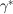) are obtained from the real-space parameters via standard crystallographic relations (see *e.g.* Giacovazzo *et al.*, 2011 ▸).

*Individual peak mapping*. This method maps the relative change in *d* spacing for each individual indexed diffraction peak [equation (8)]. It is more sensitive to anisotropic strain behavior and can reveal inter-reflection variations. This is calculated by solving for 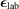 in the following equation for all indexed diffraction peaks, *n*: 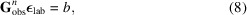
where 
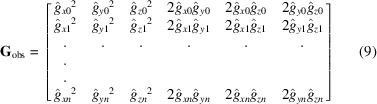

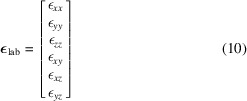
and 
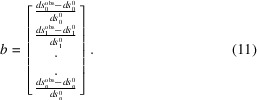
For a diffraction peak, *i*, 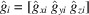 is the unit vector describing the observed diffracting plane normal and 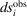 and 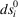 are the observed and undeformed *d* spacing, respectively.

Note that the shear strain components in equation (10) are each half of the engineering shear strain typically denoted as γ. Also note the symbols used in equations (8)–(11) follow standard conventions for strain analysis and are distinct from their usage in earlier sections describing the indexing formalism.

The numerical results of the two methods are generally consistent but can differ based on the strain anisotropy and data quality. The procedure for converting the strain tensors computed above into stress tensors using Hooke’s law and the crystal stiffness matrix is detailed in Appendix *A*[App appa]. It is important to note that when overlapping peaks from twin-related domains are excluded from the analysis (to avoid analyzing mixed strain states), the number of independent spots available for strain refinement is reduced. Consequently, the calculated 12-parameter strain tensors for these specific grains may have higher uncertainty and should be interpreted with appropriate caution.

### 3.8. Grain tracking

Analysis can also be run in a different mode where the indexing step is skipped. In case the same volume of the sample is repeatedly interrogated, the knowledge of a previous microstructure can be used to inform and accelerate the reconstruction. Starting from peak search and transform results, the framework will read the previous microstructure information, look for diffraction signal only from the grains detected in the previous microstructure, and save the optimized parameters of the grains according to the new diffraction peaks. However, this can have the disadvantage that only the same grains detected in the previous microstructure can be detected, which can be problematic in case new grains are present or if some grains split into multiple new ones.

Another way to track grains between sample states is to match grains after conducting independent analyses using user-determined criteria. This is described in more detail by Park *et al.* (2021 ▸) and in Part II of this series.

## 4. NF-HEDM data reduction

While the FF-HEDM methodology detailed in Section 3 yields valuable grain-averaged properties (orientation, strain, position), NF-HEDM provides complementary, spatially resolved crystallographic orientation maps within the illuminated volume. A key strength of the integrated framework, detailed in the following sections, is its capability to utilize the results from the FF-HEDM analysis to significantly inform, constrain and accelerate the NF-HEDM reconstruction process.

### 4.1. Guess for parameters of the experimental setup

In FF-HEDM, placing an area detector far away from the sample results in diffraction signals on well separated diffraction rings, which helps in determining the parameters of the experimental setup. In NF-HEDM, however, close placement of a high-resolution detector to the sample makes it difficult to assign *hkl* to diffraction peaks. In this case, a small calibration sample with a handful of grains (typically Au cube or wire, ∼50 µm in cross section) is centered around the rotation axis, and a full HEDM dataset is collected.

To generate a guess for the projection of the rotation axis and vertical center of the beam on the detector (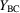, 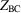), the following approach is used. Diffraction images without the beam-block, but with the calibration sample centered on the rotation axis are acquired for two ω positions separated by 180°. A starting guess for the projection of the rotation axis and vertical center of the beam on the detector (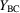, 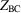) can be made by using the direct beam and attenuation signal from the sample. The user can then use the MIDAS-NF-GUI to select the same diffraction peak on different detector distances manually. Using ray-tracing, the framework computes a guess for the rotation-axis-to-detector distance (*L*). These initial guesses are then optimized, and other parameters (tilts of the detector, 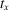, 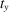 and 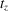) are determined by running a full reconstruction, described next. The framework assumes the tilts of the detector do not change between the multiple detector distances and that the difference in distance for the multiple detector placements is known with ∼5 µm precision. This helps constrain the parameter space during optimization (Section 4.6). Furthermore, the detector is assumed to be spatially regular. While high-resolution detectors often exhibit minor distortions (typically measured via an optical calibration grid prior to experiments), these can generally be neglected in NF-HEDM. Because the NF reconstruction relies on binarized data and spatial tolerance windows (voxels) rather than sub-pixel COM fitting, distortion amplitudes on the order of 1–2 pixels are ‘safe’ and do not severely impact the morphological reconstruction.

FF-HEDM data reduction involves computing the position of the COM of diffraction peaks and then correcting for physical imperfections of the setup (detector tilts and rotation axis misalignment). NF-HEDM, on the other hand, uses full diffraction peak shapes. In this case, applying translations and distortions to the forward simulation is computationally advantageous while keeping the observed signal as is. This results in efficient RAM and CPU utilization by storing the experimental data in a regular 3D space.

### 4.2. Image processing

(i) Raw diffraction images in NF-HEDM are first stacked together to generate a median in the rotation direction, which accounts for persistent artifacts in the images. This median is subtracted from the raw images.

(ii) The user can apply an in-image median filter with a pre-selected radius, removing a salt-and-pepper type of image noise.

(iii) The detector point-spread function is deconvolved from the signal using the Richardson–Lucy algorithm, first recommended for this purpose by Schmidt *et al.* (2008 ▸).

(iv) Diffraction peaks are then segmented first by applying a threshold, and then (optionally) the shape of the peaks is determined using an edge-detection algorithm.

(v) The segmented images are then binarized and divided into pixels with positive signal and no signal.

**Figure 5:**
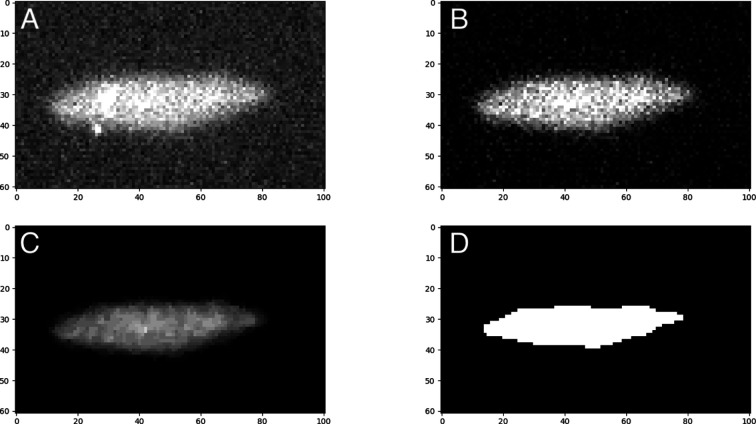
Image processing output at different stages. A zoomed-in view of the diffraction image is shown. (*a*) Raw data. (*b*) Median-corrected data. (*c*) Data after application of in-image median filter. (*d*) Binarized image after deconvolution of the point-spread function of the detector, Laplacian-of-Gaussian edge detection and application of a threshold of 5 counts.

The output of each step is shown for an example diffraction peak in Fig. 5 ▸. The 3D dataset (*y*, *z* and ω) is saved as a binary file, with each pixel occupying a single bit in memory, leading to a substantial reduction in size. (Raw images are typically 16-bit with 12-bit signal. Saving as a single bit reduces the data size by 16 times.) This 3D dataset is stored in the shared memory and shared across all the CPU cores on each computation node.

The current NF-HEDM algorithm within this framework operates on binarized diffraction data, where detector pixels are classified as either containing signal or not. This choice was made to maximize computational speed, a critical factor for real-time analysis. Consequently, the image segmentation threshold is a critical parameter that can influence the reconstructed grain morphology. Future development will focus on incorporating weighted intensity data to improve reconstruction fidelity in these challenging cases.

### 4.3. Generation of reconstruction space

The sample reconstruction space is voxelized, and a solution is computed for each voxel. Since forward simulation involves the projection of the voxel shape at different rotations and inclinations on a square grid detector, it is computationally advantageous to select the pixel shape requiring the fewest computations. In our case, a triangular voxel shape satisfies this requirement, first recommended by Suter *et al.* (2006 ▸). (Even though the detector has rectangular or square-shaped pixels, irregular overlap with voxel projections means that triangles result in the fewest computations.) These equilateral triangular voxels are packed in a hexagonal close-packed arrangement. The framework has the option to generate randomly shaped reconstruction spaces with triangular voxels. Each voxel is assumed to be comprised of a single orientation, and a single HEDM scan is one voxel high in the *z* direction.

To handle samples with complex external shapes or internal porosity, the framework allows for the application of a sample mask derived from a complementary µ-CT reconstruction, ensuring that voxels are only placed within the material itself. In this case, the user supplies a binarized tomography reconstruction image of regions inside and outside the sample. The binarized image is cropped to square dimensions, and the cropping places the rotation axis at the center of the image. The reconstruction space inside the sample is then computed after an & operation between the reconstruction space and the tomography image. The voxels in the reconstruction space are mapped to the tomography space as follows: 
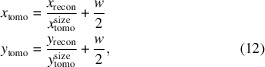
where 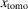 and 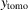 are the *x* and *y* pixel positions in the tomography reconstruction image, 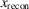 and 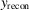 are the *x* and *y* positions in the reconstruction space, respectively, 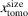 and 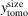 are the pixel sizes of the tomography reconstruction image in the *x* and *y* directions, respectively, and *w* is the number of pixels in the *x* or *y* direction in the square tomography image.

### 4.4. Generation of candidate orientations and forward simulation

In FF-HEDM, the framework uses information from the experiment to determine candidate orientations for indexing. The NF-HEDM geometry, however, does not yield diffraction peak locations that can *a priori* be assigned to 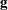. Therefore, the framework uses alternative strategies to determine candidate orientations:

(i) Use a ‘brute-force’ approach: generate equally spaced orientations in the whole fundamental region for the crystal system. Then, a forward simulation will be carried out on each orientation. For ∼2° spacing in orientation space, this procedure can lead to more than 300000 candidate orientations for cubic crystal structures.

(ii) Use the orientations obtained from FF-HEDM reconstruction of the same sample volume as candidate orientations. This leads to a significant reduction in candidate orientations, irrespective of crystal structure.

(iii) The number of candidate orientations can be further reduced by using the corresponding position of grains obtained from FF-HEDM and only trying a few of the orientations closest to the voxel in question. This way, each voxel only has a handful of possible orientations. However, this can have the following drawback: if the position from FF-HEDM has high errors, orientation selection might not include the true orientation.

(iv) Hybrid approach: try candidate orientations obtained from FF-HEDM reconstruction and try brute-force for voxels, which resulted in low-quality solutions, which is a user-defined parameter.

(v) Use orientations from vertically adjoining NF-HEDM scans on the sample as a first pass and try brute-force for low-quality solutions.

Once the candidate orientations are selected, the next step involves comparing the experiment with the forward simulation output. Since the same candidate orientations are shared between multiple voxels, the framework computes forward simulation for each candidate orientation only once, assuming a diffracting voxel located at 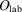. The forward simulation output is then displaced according to the position of each voxel during the ray-tracing step.

For calculating the forward simulation signal, users can choose the simulation parameters in a few different ways: (*a*) define the active area of the detector on the farthest distance and compute all diffraction peak signals; (*b*) define the *hkl*’s of interest manually – this is beneficial in case of issues with the structure factor resulting in low signal from certain *hkl*’s. The user can ignore these *hkl*’s to improve the reconstruction quality for smaller grains.

The forward simulation is saved as a shared array with 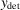, 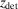 and ω position of each simulated diffraction peak at the farthest detector position, assuming no detector tilts. During comparison for each voxel in the reconstruction space, each diffraction peak position is first displaced according to the rotated position of the center of the triangle constituting the voxel; tilts and beam center corrections are then applied to calculate the simulated diffraction peak position at each detector placement.

While Friedel pairs provide a powerful constraint for candidate orientation generation in FF-HEDM, they cannot be utilized *a priori* in standard NF-HEDM due to the inherent experimental geometry. In a typical NF-HEDM setup, a vertically focused incident beam is used, and a beam-block must be placed to prevent the unattenuated direct beam from damaging the high-resolution detector. To minimize the loss of active detection area, the beam and the beam-block are typically positioned near the bottom edge of the detector. As a result, the detector only captures diffraction signals from a single hemisphere (the upper half) of the reciprocal space. Because the two diffraction events that constitute a Friedel pair propagate into opposite hemispheres, this asymmetric detector arrangement physically precludes the capture of both reflections, making Friedel pair based candidate generation impossible in the NF.

### 4.5. Ray-tracing and orientation determination

For each valid diffraction peak,^5^ the rotated triangular voxel is projected on the detector and compared with the experiment. For a successful match, all the pixels within the projected shape of the voxel need to have a positive signal (a value of 1 for binarized data) for each detector placement. This includes pixels partially within the projection. Unlike FF-HEDM, where the COM of the diffraction signal is allowed to deviate from the simulated position, NF-HEDM is stricter: signal must be observed where it is expected. The ratio of diffraction peaks where this condition is met and total simulated diffraction peaks is computed as *confidence*.

The size of voxels plays a vital role in determining the final reconstruction output. For example, if the user selects too large voxels, the condition of all pixels having a positive signal to qualify as a match would not be satisfied for small grains or voxels close to the grain boundaries. To avoid this, the spacing between voxels and the size of triangles defining the voxels are separate in the framework. The user can place voxels further apart to reduce computation time but reduce the edge length of the voxels to obtain a high-confidence reconstruction. While the physical microstructure is space-filling, the computational reconstruction grid allows users to artificially space voxels further apart (creating ‘empty’ space between queried locations). This computational strategy significantly reduces computation time, allowing for rapid, coarse preliminary reconstructions to verify data quality before running a fully dense, space-filling final reconstruction.

The framework computes the confidence for each of the candidate orientations for a voxel. The actual orientation for the voxel is not necessarily equal to the candidate orientation but is close to it. All candidate orientations are not optimized, which would be computationally expensive. All the candidate orientations for which the confidence exceeds a user-selected value are then fed to the next part: optimizing the orientation. The orientation is represented as Euler angles during optimization. (Euler angles suffer from the infamous gimbal lock, but they are computationally advantageous during optimization because the three components can be changed independently.) The optimized orientation, which yields the highest confidence, is selected as the solution for the voxel. It has been observed that voxels close to the grain boundaries can sometimes have multiple orientations with similar confidence. Thus, the framework can save a user-selected number of next-best orientations for each voxel.

### 4.6. Optimization of the experimental setup

After obtaining an initial guess of the parameters of the experimental setup (Section 4.1), the user can optimize the parameters using an initial pass reconstruction. Optimizing the parameters involves refining, for *n* detector distances, *n* rotation-axis-to-detector distances, *n* beam centers (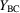 and 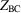), tilts (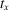, 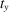 and 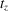) and crystallographic orientation of user-selected voxels to achieve the highest total confidence. The selection of voxels is essential here to get the best parameters characterizing the setup. For example, suppose the user selects a single voxel away from the rotation axis for optimization. In that case, the convolution between beam centers and tilts can lead to high confidence at the selected voxel but low confidence overall in the reconstruction. Thus, the recommended strategy is to choose multiple voxels (this number is usually between 6 and 20) spread around the rotation axis and optimize all the voxels together.

### 4.7. Grain determination

The framework computes a solution to each voxel independently. The user can select a misorientation value between neighbors to compute grains in 2D (using a single reconstruction) or 3D (using multiple reconstructions from consecutive layers in a sample). For further processing, the output can also be pipelined into post-processing software such as *DREAM.3D* (Groeber & Jackson, 2014 ▸).

## 5. Computational implementation and availability

The methodological framework described in this paper is implemented in the open-source software package *MIDAS*.

*Language and dependencies*. The core logic is implemented in Python 3, with computationally intensive routines written in C for performance. The code leverages standard scientific libraries such as *NumPy*, *SciPy* and the *NLopt* library for optimization (Johnson, 2023 ▸).

*Availability and documentation*. *MIDAS* is open-source and is publicly available on GitHub at https://github.com/marinerhemant/MIDAS, under an MIT license. Full documentation, including installation guides and tutorials, is provided with the repository.

*Community validation*. The utility and adequacy of this framework have been demonstrated through its successful use by numerous independent research groups at the APS and other facilities, as shown by publications such as Amirrahmat *et al.* (2020 ▸) and Dixit *et al.* (2022 ▸).

## 6. Summary

This paper has detailed a complete and unified methodological framework for the robust and efficient inversion of HEDM diffraction data from both near-field and far-field geometries. We have presented the mathematical formalisms, physical models and key algorithmic innovations, including a generalized detector distortion model, an efficient indexing strategy based on Friedel pair constraints and a decoupled refinement scheme. The framework is designed to be computationally efficient for the large datasets generated by modern synchrotrons. This paper provides the necessary foundational principles for the rigorous experimental validation and derivation of best practices presented in Part II of this series.

## Figures and Tables

**Table 1:** Summary of notation used in the paper

Symbol	Meaning	First use
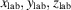	Laboratory coordinate system axes	Section 2.2
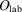	Laboratory coordinate system origin	Section 2.2
ω	Sample rotation angle about 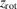	Section 2.1
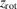	Sample rotation axis	Section 2.1
Ω	Wedge angle between 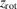 and 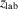	Section 2.2
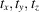	Detector rotations about laboratory axes	Section 2.2
*L*	Rotation-axis-to-detector distance	Section 2.2
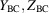	Projection of 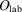 onto detector (beam center)	Section 3.1
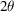	Diffraction angle (Bragg angle ×2)	Section 3.3
η	Azimuthal angle on a Debye–Scherrer ring	Section 3.3
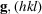	Diffraction plane normal, Miller indices	Section 3.3
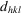	Interplanar spacing for (*hkl*)	Section 3.3
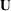	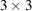 orientation matrix (grain to laboratory)	Section 3.7
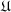	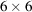 Mandel–Voigt transformation matrix	Appendix *A*[App appa]
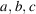	Unit-cell edge lengths	Section 3.7
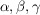	Unit-cell inter-axial angles	Section 3.7
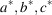	Reciprocal-lattice edge lengths	Section 3.7
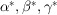	Reciprocal-lattice inter-axial angles	Section 3.7
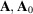	Real-space and unstrained orthogonalization matrices	Section 3.7
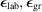	Strain tensor in laboratory/grain frames	Section 3.7
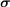	Stress tensor	Appendix *A*[App appa]
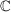	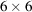 elastic stiffness matrix	Appendix *A*[App appa]
*I*	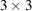 identity matrix	Section 3.7
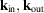	Incident and scattered wavevectors	Section 3.3
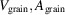	Grain volume/cross-sectional area	Section 3.3
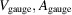	Illuminated sample volume/area	Section 3.3
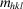	(*hkl*) plane multiplicity	Section 3.3
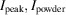	Peak/powder intensity	Section 3.3
MinNrSols, Confidence	Indexing quality thresholds	Section 3.4

## Footnotes

1 There can be a total of four diffraction peaks, which form two distinct Friedel pairs. Detailed description can be found in the work of Sharma *et al.* (2012*a* ▸).

2 Similar grain sizes can yield very different integrated intensities depending on the azimuthal angle, structure factor and Lorentz–polarization factors. *MIDAS* computes the originating grain size after correcting for all these factors, as described in Section 3.3.

3 This depends on the multiplicity of the diffraction ring selected to generate candidate orientations, the fraction of diffraction peaks in that ring selected for processing and tolerance to reconstruction errors.

4 One example of twin relationship for f.c.c. (face-centered cubic) structures is a rotation of 60° around the 〈111〉 axis.

5 A hit on the active area of the detector within the experiment rotation range qualifies as a valid diffraction peak.

## Appendix A Stress calculation

The strain output from the framework can be vectorized as the following (this is often referred to as Voigt–Mandel notation):

Hooke’s law converts the measured strain into stresses:

In this equation,  is stress and  is the stiffness matrix truncated to a 6 × 6 matrix (Hosford, 1993 ▸). Using the *U* matrix introduced in Section 3.7, we can also define  that transforms the vectorized strain and stress from the laboratory (or sample, if the *U* matrix accounts for the laboratory-to-sample-frame transformation) to the crystal frame:

Finally, the stress in the crystal frame, , can be computed based on strain output using

Notably, the stress in the laboratory frame can be computed using the following:

## Appendix B Notation summary

Table 1 ▸ summarizes the mathematical notation used throughout the paper for ease of reference. Symbols appearing only once in a specific equation are omitted.

## References

[bb1] Ahrens, J., Geveci, B. & Law, C. (2005). *Visualization Handbook*, pp. 717–731. Elsevier.

[bb2] Amirrahmat, S., Imseeh, W. H., Alshibli, K. A., Kenesei, P., Jarrar, Z. A. & Sharma, H. (2020). *J. Geotech. Geoenviron. Eng.* **146**, 04020027.

[bb3] Bernier, J. V., Barton, N. R., Lienert, U. & Miller, M. P. (2011). *J. Strain Anal. Eng. Des.* **46**, 527–547.

[bb4] Bonnin, A., Wright, J. P., Tucoulou, R. & Palancher, H. (2014). *Appl. Phys. Lett.* **105**, 084103.

[bb5] Borbely, A., Renversade, L., Kenesei, P. & Wright, J. (2014). *J. Appl. Cryst.* **47**, 1042–1053.

[bb6] Busing, W. R. & Levy, H. A. (1967). *Acta Cryst.* **22**, 457–464.

[bb7] Cherukara, M. J., Cha, W. & Harder, R. J. (2018). *Appl. Phys. Lett.* **113**, 203101.

[bb8] Dixit, M. B., Vishugopi, B. S., Zaman, W., Kenesei, P., Park, J.-S., Almer, J., Mukherjee, P. P. & Hatzell, K. B. (2022). *Nat. Mater.* **21**, 1298–1305. 10.1038/s41563-022-01333-y 36050382

[bb9] Giacovazzo, C., Monaco, H. L., Artioli, G., Viterbo, D., Milanesio, M., Gilli, G., Gilli, P., Zanotti, G., Ferraris, G. & Catti, M. (2011). *Fundamentals of Crystallography.* Oxford University Press.

[bb10] Groeber, M. A. & Jackson, M. A. (2014). *Integr. Mater. Manuf. Innov.* **3**, 56–72.

[bb11] Grosse-Kunstleve, R. W. (2023). *SgInfo*. https://cci.lbl.gov/sginfo/.

[bb12] Hosford, W. F. (1993). *The Mechanics of Crystals and Textured Polycrystals.* Oxford Engineering Science Series. Oxford University Press.

[bb13] Hubbell, J. H. & Seltzer, S. M. (2024). *NIST Standard Reference Database 126.*https://www.nist.gov/pml/x-ray-mass-attenuation-coefficients.

[bb14] Jakobsen, B., Poulsen, H. F., Lienert, U., Almer, J., Shastri, S. D., Sørensen, H. O., Gundlach, C. & Pantleon, W. (2006). *Science* **312**, 889–892. 10.1126/science.1124141 16690859

[bb15] Johnson, Q. C., Kenesei, P., Petruzza, S., Plumb, J., Sharma, H., Park, J. S., Marsden, E., Matheson, K., Czabaj, M. W. & Spear, A. D. (2023). *Mater. Charact.* **195**, 112477.

[bb16] Johnson, S. G. (2023). *NLopt*. https://github.com/stevengj/nlopt.

[bb17] Kim, J., Hayashi, Y. & Yabashi, M. (2023). *J. Appl. Cryst.* **56**, 1416–1425.

[bb18] Larson, B. C., Yang, W., Ice, G. E., Budai, J. D. & Tischler, J. Z. (2002). *Nature* **415**, 887–890. 10.1038/415887a 11859363

[bb19] Lee, J. H., Aydıner, C. C., Almer, J., Bernier, J., Chapman, K. W., Chupas, P. J., Haeffner, D., Kump, K., Lee, P. L., Lienert, U., Miceli, A. & Vera, G. (2008). *J. Synchrotron Rad.* **15**, 477–488. 10.1107/S090904950801755X 18728319

[bb20] Li, W., Sharma, H., Kenesei, P., Ravi, S., Sehitoglu, H. & Bucsek, A. (2023). *J. Mater. Res.* **38**, 165–178.

[bb21] Liu, Z., Sharma, H., Park, J.-S., Kenesei, P., Miceli, A., Almer, J., Kettimuthu, R. & Foster, I. (2022). *IUCrJ* **9**, 104–113. 10.1107/S2052252521011258 PMC8733885 35059215

[bb22] Ludwig, W., Reischig, P., King, A., Herbig, M., Lauridsen, E. M., Johnson, G., Marrow, T. J. & Buffière, J. Y. (2009). *Rev. Sci. Instrum.* **80**, 033905. 10.1063/1.3100200 19334932

[bb23] Maddali, S., Park, J. S., Sharma, H., Shastri, S., Kenesei, P., Almer, J., Harder, R., Highland, M. J., Nashed, Y. & Hruszkewycz, S. O. (2020). *Phys. Rev. Appl.* **14**, 024085.

[bb24] Marone, F. & Stampanoni, M. (2012). *J. Synchrotron Rad.* **19**, 1029–1037. 10.1107/S0909049512032864 PMC3480277 23093766

[bb25] Moscicki, M., Kenesei, P., Wright, J., Pinto, H., Lippmann, T., Borbély, A. & Pyzalla, A. R. (2009). *Mater. Sci. Eng. A* **524**, 64–68.

[bb26] Nygren, K. E., Pagan, D. C., Bernier, J. V. & Miller, M. P. (2020). *Mater. Charact.* **165**, 110366.

[bb27] Oddershede, J., Camin, B., Schmidt, S., Mikkelsen, L. P., Sørensen, H. O., Lienert, U., Poulsen, H. F. & Reimers, W. (2012). *Acta Mater.* **60**, 3570–3580.

[bb28] Offerman, S. E. & Sharma, H. (2010). In *In-situ Studies with Photons, Neutrons and Electrons Scattering*, edited by T. Kannengiesser *et al.* Berlin: Springer.

[bb29] Pagan, D. C., Bernier, J. V., Dale, D., Ko, J. Y., Turner, T. J., Blank, B. & Shade, P. A. (2018). *Scr. Mater.* **142**, 96–100.

[bb30] Park, J.-S., Sharma, H. & Kenesei, P. (2021). *J. Synchrotron Rad.* **28**, 1786–1800. 10.1107/S1600577521008286 34738932

[bb32] Poulsen, H. (2004). *Three-Dimensional X-ray Diffraction Microscopy*, Vol. 205. Springer Tracts in Modern Physics. Berlin: Springer.

[bb33] Poulsen, H. F. (2003). *Philos. Mag.* **83**, 2761–2778.

[bb34] Schmidt, S., Olsen, U. L., Poulsen, H. F., Sørensen, H. O., Lauridsen, E. M., Margulies, L., Maurice, C. & Juul Jensen, D. (2008). *Scr. Mater.* **59**, 491–494.

[bb35] Shade, P. A., Blank, B., Schuren, J. C., Turner, T. J., Kenesei, P., Goetze, K., Suter, R. M., Bernier, J. V., Li, S. F., Lind, J., Lienert, U. & Almer, J. (2015). *Rev. Sci. Instrum.* **86**, 093902. 10.1063/1.4927855 26429452

[bb36] Sharma, H., Huizenga, R. M. & Offerman, S. E. (2012*a*). *J. Appl. Cryst.* **45**, 693–704.

[bb37] Sharma, H., Huizenga, R. M. & Offerman, S. E. (2012*b*). *J. Appl. Cryst.* **45**, 705–718.

[bb50] Sharma, H., Park, J.-S., Shastri, S. & Kenesei, P. (2026). *Acta Cryst.* A**82**, 305–320. 10.1107/S2053273326004018 PMC13325190 42206381

[bb38] Sharma, H., Wattjes, A. C., Amirthalingam, M., Zuidwijk, T., Geerlofs, N. & Offerman, S. E. (2009). *Rev. Sci. Instrum.* **80**, 123301. 10.1063/1.3262501 20059134

[bb39] Shastri, S. D. (2004). *J. Synchrotron Rad.* **11**, 150–156. 10.1107/S0909049503023586 14960779

[bb40] Shastri, S. D., Almer, J., Ribbing, C. & Cederström, B. (2007). *J. Synchrotron Rad.* **14**, 204–211. 10.1107/S0909049507003962 17317922

[bb41] Shastri, S. D., Fezzaa, K., Mashayekhi, A., Lee, W.-K., Fernandez, P. B. & Lee, P. L. (2002). *J. Synchrotron Rad.* **9**, 317–322. 10.1107/s0909049502009986 12200577

[bb42] Simons, H., King, A., Ludwig, W., Detlefs, C., Pantleon, W., Schmidt, S., Stöhr, F., Snigireva, I., Snigirev, A. & Poulsen, H. F. (2015). *Nat. Commun.* **6**, 6098. 10.1038/ncomms7098 PMC4354092 25586429

[bb43] Suter, R. M., Hennessy, D., Xiao, C. & Lienert, U. (2006). *Rev. Sci. Instrum.* **77**, 123905.

[bb44] Wong-Ng, W., Siegrist, T., DeTitta, G. T., Finger, L. W., Evans, H. T., Gabe, E. J., Enright, G. D., Armstrong, J. T., Levenson, M., Cook, L. P. & Hubbard, C. R. (2001). *J. Res. Natl Inst. Stand. Technol.* **106** (6). 10.6028/jres.106.058 PMC4865310 27500067

[bb45] Wright, J. P. (2020). *FABLE-3DXRD/ImageD11*. https://github.com/FABLE-3DXRD/ImageD11.

[bb46] Wright, J. P., Giacobbe, C. & Lawrence Bright, E. (2022). *Crystals* **12**, 255.

